# Development of Lipid Nanoparticle Formulation for the Repeated Administration of mRNA Therapeutics

**DOI:** 10.34133/bmr.0017

**Published:** 2024-05-22

**Authors:** Yeji Lee, Michaela Jeong, Gyeongseok Lee, Jeongeun Park, Hyein Jung, Seongeun Im, Hyukjin Lee

**Affiliations:** College of Pharmacy, Graduate School of Pharmaceutical Sciences, Ewha Womans University, Seoul 03760, Republic of Korea.

## Abstract

During the COVID-19 pandemic, mRNA vaccines emerged as a rapid and effective solution for global immunization. The success of COVID-19 mRNA vaccines has increased interest in the use of lipid nanoparticles (LNPs) for the in vivo delivery of mRNA therapeutics. Although mRNA exhibits robust expression profiles, transient protein expression is often observed, raising uncertainty regarding the frequency of its administration. Additionally, various RNA therapeutics may necessitate repeated dosing to achieve optimal therapeutic outcomes. Nevertheless, the impact of repeated administrations of mRNA/LNP on immune responses and protein expression efficacy remains unclear. In this study, we investigated the influence of the formulation parameters, specifically ionizable lipids and polyethylene glycol (PEG) lipids, on the repeat administration of mRNA/LNP. Our findings revealed that ionizable lipids had no discernible impact on the dose-responsive efficacy of repeat administrations, whereas the lipid structure and molar ratio of PEG lipids were primary factors that affected mRNA/LNP performance. The optimization of the LNP formulation with PEG lipid confirmed the sustained dose-responsive efficacy of mRNA after repeated administrations. This study highlights the critical importance of optimizing LNP formulations for mRNA therapeutics requiring repeated administrations.

## Introduction

Since the onset of the COVID-19 pandemic, there has been an accelerated development and global deployment of mRNA/lipid nanoparticle (mRNA/LNP) vaccines [1,2]. Consequently, there has been an increase in interest and research pertaining to LNPs, because of their pivotal role in enhancing the in vivo delivery of mRNA. LNPs are efficient nonviral gene delivery carriers composed of four primary lipid constituents: ionizable lipids, phospholipids, cholesterol, and polyethylene glycol (PEG) lipids. Ionizable lipids, characterized by a tertiary amine structure, enable RNA encapsulation and facilitate its transport into the cytoplasm [3,4]. Phospholipids and cholesterol contribute to LNP stabilization and aid endosome escape, a critical factor that ensures LNP efficacy. PEG lipids are situated on the LNP surface, mitigating opsonization and thereby extending their half-life and circulation within the body [5,6].

Because mRNA is easily degraded and disappears in the body, repeated administration is necessary, which can enhance immunological responses and provide therapeutic advantages [7]. The optimization of mRNA/LNP formulations is crucial to enable repeated administrations. Currently, US Food and Drug Administration (FDA)-approved drugs such as Onpattro are administered repeatedly over 18 months via intravenous injection at 3-week intervals. Similarly, COVID-19 vaccines including Spikevax and Comirnaty necessitate repeated administrations at 3- to 4-week intervals [8–10]. Moreover, preclinical studies of mRNA/LNP therapies predominantly use repeated dosages. For instance, protein replacement treatments developed for hemophilia require repeated intravenous administration every 5 to 10 days, whereas those designed for metabolic diseases use various administration intervals ranging from 3 to 10 days [11–15]. Gene editing treatments using CRISPR/Cas9 mRNA, adenine base editor mRNA, and cytidine base editor mRNA exhibit varying usage intervals, spanning from 3 days to weekly or every 2 weeks. Nevertheless, the impact of repeated mRNA/LNP administrations on immune responses and protein expression efficacy remains unclear [16–18].

**Fig. 1.**
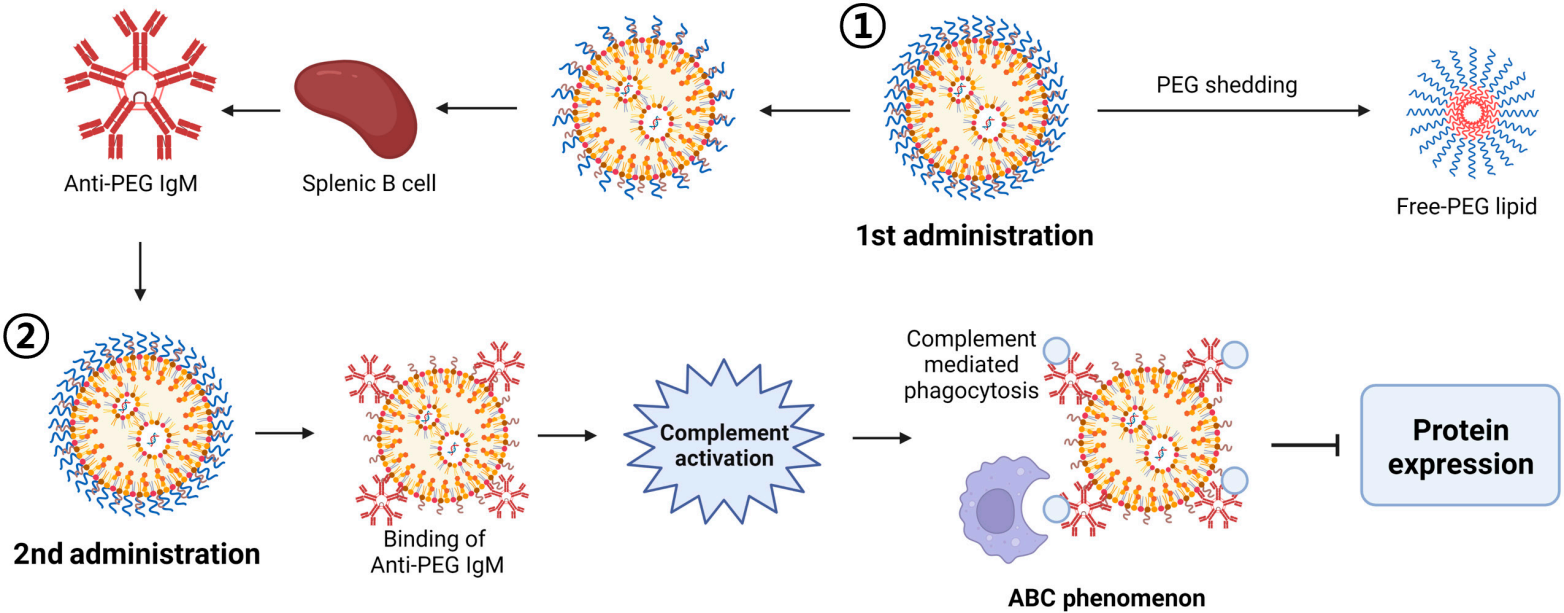
ABC phenomenon and its effects after the repeated administration of mRNA/LNP. When LNP encapsulated with mRNA is administered for the first time, PEG is sheathed in the body, and the remaining LNP induces anti-PEG IgM production by splenic B cells. If mRNA/LNP is administered a second time while this antibody remains in the body, anti-PEG IgM binds to the surface of the LNP, causing complement activation. Therefore, LNP is removed through phagocytosis, which inhibits mRNA expression.

Various studies have examined the repeated administration of PEGylated liposomes and PEGylated nanoparticles (PEG-NPs) [19–22]. PEG lipids have been recognized for their ability to extend the serum half-life of LNPs by mitigating opsonization [23,24]. However, the administration of PEG-NPs elicited the production of anti-PEG immunoglobulin M (IgM) antibodies in vivo [25], which are generated through the proliferation and differentiation of specific B cells in the splenic marginal zone [26]. Upon subsequent injection, anti-PEG IgM triggers complement activation, culminating in the accelerated blood clearance (ABC) phenomenon [27], which rapidly removes subsequently administered PEG-NPs, leading to diminished drug efficacy due to reduced nanoparticle concentration in the bloodstream [28–31]. Consequently, numerous studies have endeavored to alleviate the ABC phenomenon [32]. Among these strategies, shortening the length of PEG lipids was investigated to expedite PEG shedding and reduce anti-PEG IgM production, thereby ameliorating the ABC phenomenon (Fig. 1) [33]. This highlights the paramount importance of optimizing repeated administration protocols for the future development and formulation of LNPs. Further research is necessary to evaluate the impact of PEG lipid type and quantity on protein expression efficacy and gene editing via repeated administrations of mRNA/LNP. Presently, investigations into the consequences of the ABC phenomenon on immunological responses and protein expression have been limited.

In this study, we examined the influence of ionizable lipids and PEG lipids on mRNA/LNP formulations during repeated administrations. Our findings indicate that ionizable lipids do not exert a discernible impact on repeated administrations, whereas the lipid structure and molar ratio of PEG lipids have a important role. Notably, the formation of anti-PEG IgM appears to be influenced by PEG attached to LNPs rather than free-PEG. In the body, PEG-incorporated LNPs are perceived as large PEG entities, potentially provoking antibody production. This phenomenon results in diminished protein expression upon the repeated administrations of mRNA/LNP. Specifically, repeated systemic injections of firefly luciferase (fLuc) mRNA led to a large decline in luminescent expression in the liver. Likewise, gene editing outcomes following repeated administration varied depending on the specific PEG utilized. Our study clearly shows the critical importance of selecting the structure and molar ratio of PEG lipids when formulating LNPs for repeated administrations.

## Materials and Methods

### Materials

1,2-Distearoyl-sn-glycero-3-phosphocholine (DSPC), 1,2-dioleoyl-snglycero-3-phosphoethanolamine (DOPE), 1,2-dimyristoyl-rac-glycero-3-methoxypolyethylene glycol-2000 (880151P), and C16-PEG 2000 ceramide (880180) were purchased from Avanti Polar Lipids. SM-102 [(heptadecan-9-yl 8-((2-hydroxyethyl)(6-oxO-6-(undecyloxy) hexyl) amino) octanoate), CAS: 2089251-47-6] was purchased from SINOPEG. Cholesterol (C8667) and PEG 10k (8.21881) and PEG 35k (8.18892.1000) were purchased from Sigma-Aldrich (St. Louis, MO, USA). d-Luciferin was purchased from Promega (P1043). The RNA encapsulation efficiency was quantified using a Quant-iT Ribogreen Assay (Life Technologies, USA). Analytical flow cytometry was performed on the NovoCyte 2060R (ACEA Biosciences Inc., San Diego, CA, USA) at Ewha Drug Development Research Core Center. Reporter mRNAs (fLuc mRNA, Cre mRNA, and hEPO mRNA) used in this study were purchased from TriLink BioTechnologies (L-7602, L-7211, L-7209; San Diego, CA, USA). hEPO enzyme-linked immunosorbent assay (ELISA) kits were purchased from R&D Systems (DEP00; Minneapolis, MN, USA). MCP-1 ELISA kits were purchased from Thermo Fisher Scientific (BMS281; Waltham, MA, USA). Liberase Research Grade (5401127001), deoxyribonuclease (DNase) I (4716728001), and hyaluronidase (H350.6) were purchased from Sigma-Aldrich (St. Louis, MO, USA). Mouse complement C5a ELISA kits were purchased from Abcam (ab193718), and a mouse anti-PEG IgM ELISA was purchased from Life Diagnostics (PEGM-1).

### Preparation of LNPs

To formulate LNPs, the ethanol phase containing lipid components and an aqueous phase containing RNA were mixed via a microfluidic mixing system (Ignite, PNI, Canada). mRNA-loaded 244-cis and SM-102 LNPs were formulated via a microfluidic device as previously reported [34]. Ionizable lipids (244cis, SM-102), cholesterol, helper lipids (DOPE, DSPC), and PEG lipid (C16-PEG 2000 ceramide, DMG-PEG 2000) were dissolved in ethanol. The molar ratio of the lipid components was 244-cis:DOPE:cholesterol:C16-PEG ceramide = 26.5:20:52:1.5 or 26.5:20:52.4:1.1 and SM-102:DSPC:cholesterol:DMG-PEG 2000 = 50:10:38.5:1.5. mRNA was diluted using phosphate-buffered saline (PBS) and 10.0 mM citrate buffer, and the volume ratio of PBS to 10.0 mM citrate buffer was 2:1. The final ionizable lipid:RNA weight ratio was 10:1. The formulation rate and final volume ratio of the lipid phase:RNA phase was 1:3. LNPs were formulated by microfluidic mixing of the prepared solutions at a 12 ml/min flow rate. The resulting LNPs were diluted in a 40-fold volume of 1× PBS (SH30028.02, Cytiva) and concentrated via ultrafiltration (Amicon Ultra-15 Centrifugal Filter Unit, UFC9010).

### Characterization of LNPs

A Zetasizer Nano ZS90 (Malvern Instruments, Malvern, UK) at Ewha Drug Development Research Core Center was used to measure the mean particle size and polydispersity index (PDI) of LNPs diluted to an RNA dose of 0.001 mg ml^−1^ with PBS. RNA therapeutic encapsulation efficiency was measured by a Quant-iT Ribogreen Assay (Life Technologies).

### In vivo mRNA delivery

LNP at a dose of 0.5 mg/kg based on hEPO mRNA was administrated intravenously into 7-week-old BALC/c mice (Orient Bio), and protein expression was investigated after 6 h using a hEPO ELISA kit. The blood was collected via cheek bleeding, and the serum was separated from the blood. Serum was diluted 5000 times and then added to the hEPO ELISA kit. The second administration was performed the same way as the first, 7 days later. Anti-PEG IgM was analyzed before the second administration, and complement C5a was analyzed 6 h after the second administration. Serum was diluted 150 or 10 times and then used for anti-PEG IgM ELISA or complement C5a ELISA, respectively.

LNP at a dose of 0.1 mg/kg based on hEPO mRNA was administrated intravenously into 7-week-old BALC/c mice (Orient Bio), and initial immune responses were investigated after 3 h using the MCP-1 ELISA kit. The blood was collected via cheek bleeding, and the serum was separated from the blood. Undiluted serum was used for the MCP-1 ELISA kit.

Seven-week-old female C57BL/6 mice weighing 18 to 20 g were purchased from Orient Bio (Seongnam, South Korea). Mice were administered fLuc mRNA/loaded LNPs via retro-orbital injection at an mRNA dose of 0.1 mg/kg. Six hours later, mice were injected intraperitoneally with 200 μl of 30 mg/ml d-luciferin (VivoGlo Luciferin; Promega, Madison, WI, USA). Twenty minutes later, mice were euthanized by CO_2_ inhalation. Luminescence was confirmed by an IVIS Lumina system (IVIS Lumina III imaging system, PerkinElmer, Waltham, MA, USA) at the Ewha Drug Development Research Core Center. This study was approved by the Institutional Animal Care and Use Committee at Ewha Womans University (EWHA IACUC, past-23-048).

### Cell-specific delivery of LNPs to LSL-tdTomato mice

LSL-tdTomato Ai14 mice (#:007914) were purchased from Jackson Laboratory. Eight-week-old female mice were injected intravenously at a dose of 0.01 mg/kg of Cre mRNA/LNP. After 2 days, mice were anesthetized by isoflurane and the liver was perfused with 25 ml of 1× PBS. TdTomato fluorescence in five organs (heart, lungs, liver, spleen, and kidney) was confirmed using an IVIS Lumina system. Livers were collected in RPMI 1640 (11875093) medium and digested with 10 mg of Liberase, 10 mg of hyaluronidase, and 50 U of DNase I at 37 °C for 30 min. Then, the liver solution was filtered using a 40-μm filter. A cell pellet was obtained by centrifuging (300*g*) for 10 min. The supernatant was removed, and the cell pellet was resuspended with cell staining buffer (BioLegend). After that, the cells were incubated with antibodies in the dark at 4 °C for 20 min. The antibodies used were allophycocyanin (APC)-labeled anti-CD31 (BioLegend), fluorescein isothiocyanate (FITC)-labeled anti-CD45 (BioLegend), and phycoerythrin (PE)/cy7-labeled anti-CD68 (BioLegend). The liver cells were analyzed using the NovoCyte 2060R (ACEA Biosciences Inc., San Diego, CA, USA). A representative flow cytometry gate strategy is shown in Fig. S6

All data are shown as the means ± SD. The data were analyzed by one-way analysis of variance (ANOVA) and two-way ANOVA. A *P* value of <0.05 was considered significant.

## Results and Discussion

### The structure of the PEG lipid affects repeated doses of SM-102 LNPs

**Fig. 2.**
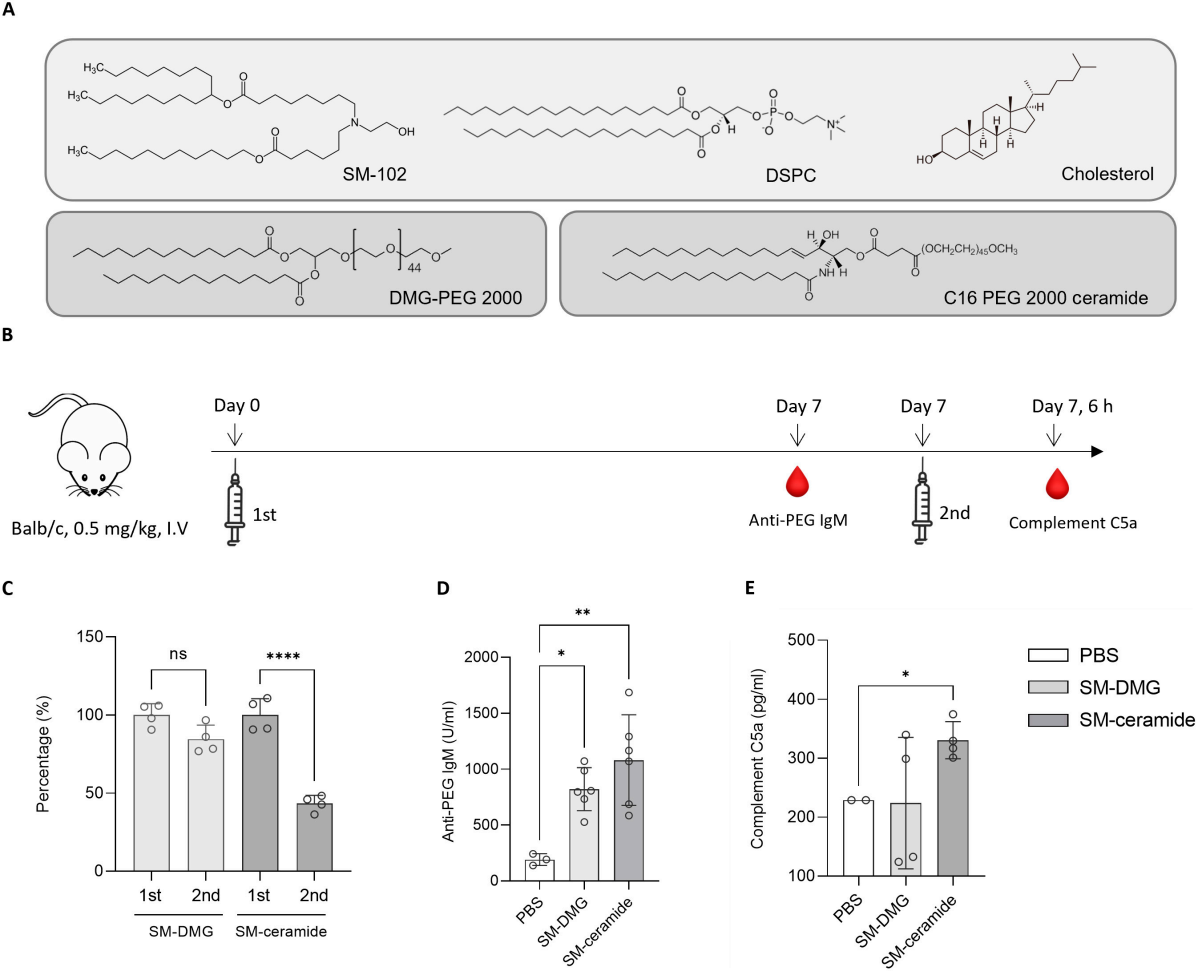
Evaluation of the repeated administration on changes in SM-102 PEG lipid. (A) Components of SM-102 LNP. SM-102 LNP contains SM-102 ionizable lipid, DSPC, cholesterol, and DMG-PEG 2000. In this study, DMG-PEG 2000 in SM-102 was changed to C16 PEG 2000 ceramide. (B) Experimental schedule for the repeated LNP dosage. A dose of 0.5 mg/kg LNP encapsulating hEPO mRNA was delivered intravenously into a 7-week-old female Balb/c mouse and evaluated 6 h later using the hEPO ELISA kit. The second administration was carried out in the same manner as the first, 7 days later. Anti-PEG IgM was measured before the second injection, and complement C5a was measured in the plasma 6 h later. (C) Changes in mRNA efficiency following the repeated administration of LNP. The protein expression efficacy of the second administration was quantified using hEPO ELISA, and it was converted into a percentage based on the first administration. (D) Concentration of anti-PEG IgM 1 week after LNP administration. It was confirmed that the antibody concentration of C16 PEG ceramide LNP was higher than that of DMG-PEG-LNP. (E) Complement C5a concentration after the second administration of LNP. Complement activity appears to be increasing as the complement C5a concentration in the SM-ceramide group slightly increases.

Spikevax, Moderna's FDA-approved COVID-19 vaccine, contains SM-102 ionizable lipid, DSPC, cholesterol, and DMG-PEG (1,2-dimyristoyl-rac-glycero-3-methoxypolyethylene glycol-2000) (Fig. 2A). To investigate the effect of repeated administrations, the PEG lipid of SM-102 LNP was changed to C16 PEG ceramide {N-palmitoyl-sphingosine-1-succinyl [methoxy (polyethylene glycol)2000]}, and the concentration of human erythropoietin (hEPO) protein was analyzed after each administration (Fig. 2A). The physical properties of the LNPs are shown in Table S1. To validate the ABC phenomenon after repeated administrations of LNPs, the concentrations of anti-PEG IgM and complement C5a induced by LNPs were analyzed by ELISA. Complement C5a is a terminal product of complement activation that can be used to determine whether complement activation has occurred [35,36]. LNP at a dose of 0.5 mg/kg based on hEPO mRNA was administrated intravenously into 7-week-old Balb/c mice, and protein expression was investigated after 6 h using a hEPO ELISA kit. The second administration was performed the same way as the first, 7 days later. When PEG-NPs are injected, anti-PEG IgM is generated and the concentration peaks 7 days after the administration [22,37]. In the case of complement C5a, the response was activated within 4 to 10 h after injection [36,38]. Therefore, anti-PEG IgM was analyzed before the second administration and complement C5a was analyzed 6 h after the second administration. The repeated administration schedule is shown in Fig. 2B. The protein expression efficacy of the second administration of LNPs was calculated as a percentage based on the hEPO concentration of the first administration (Fig. 2C). Figure S1 provides details on the hEPO concentration. As a result, DMG-PEG LNP maintained 84.5% of the first dose in the second dose but decreased to 43.4% in the case of C16 PEG ceramide. It appears that the structure of PEG lipid affects protein expression after repeated administrations. As a result of analyzing the concentration of anti-PEG IgM 1 week after LNP administration, the antibody concentration of C16 PEG ceramide LNP was higher than that of DMG-PEG-LNP (Fig. 2D). In addition, as the concentration of complement C5a slightly increased after the second administration of C16 PEG ceramide LNP, complement activity increased and the ABC phenomenon occurred (Fig. 2E).

### Anti-PEG IgM is produced by PEG linked to LNP rather than free-PEG lipid, and LNP-PEG is considered a large PEG

**Fig. 3.**
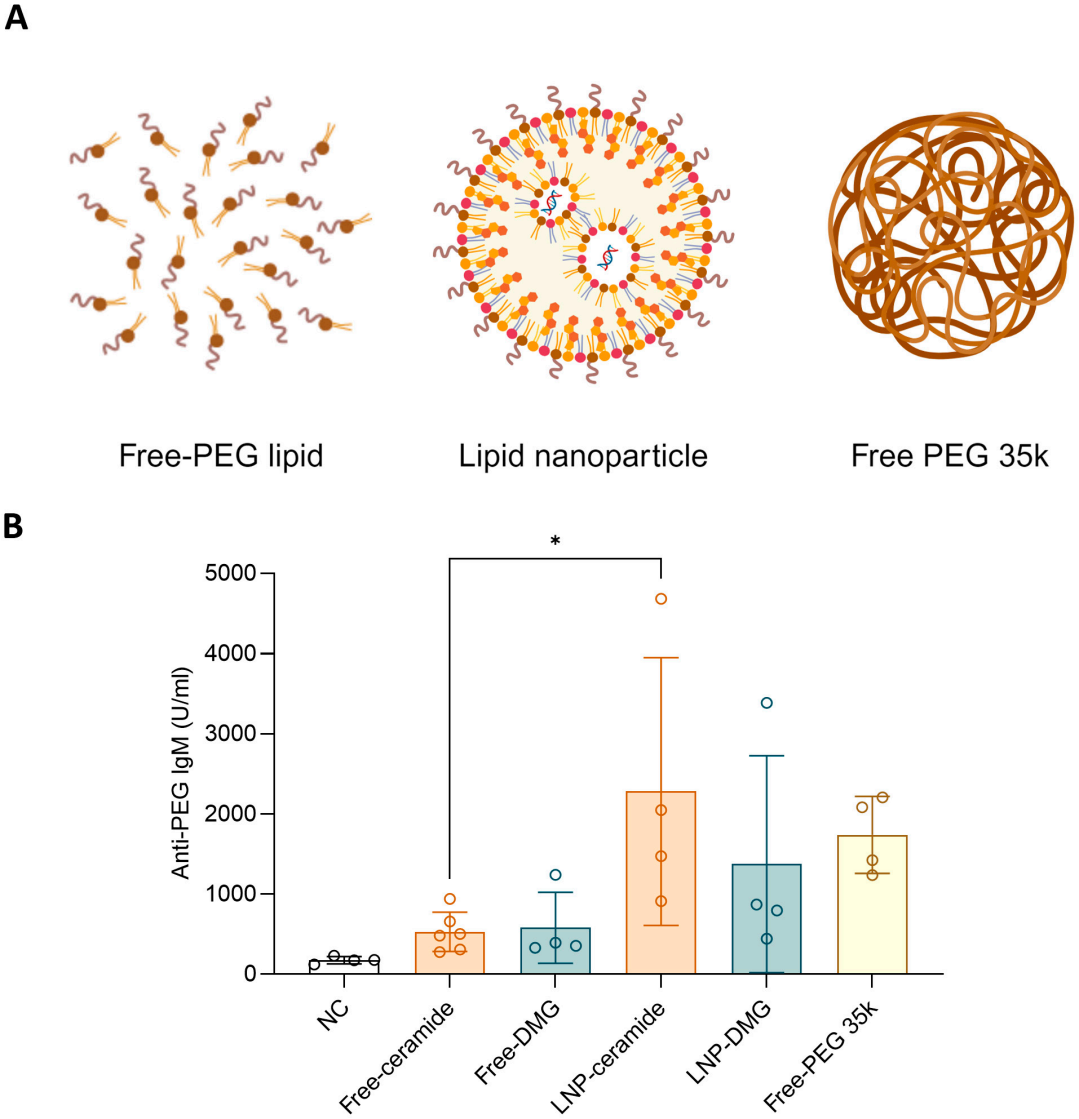
Comparison of anti-PEG antibody formation between free-PEG lipid, PEG incorporated LNP, and high molecular weight PEG (35k). (A) A dose of 0.5 mg/kg of mRNA/244-cis LNP (LNP-ceramide group, LNP-DMG group) and the same amount of free-PEG lipids (2k PEG ceramide group, 2k PEG DMG group) and 35k free-PEG lipids were injected intravenously into Balb/c mice. (B) Seven days after administration, anti-PEG IgM was measured by ELISA.

We hypothesized that anti-PEG IgM in vivo is produced by PEG lipid linked to mRNA/LNP rather than free-PEG lipid. The same mole of free-C16 PEG ceramide PEG lipid (free-ceramide group) as mRNA/244-cis LNP (LNP-ceramide group) at a dose of 0.5 mg/kg mRNA was intravenously injected into Balb/c mice (Fig. 3A). Anti-PEG IgM was analyzed by ELISA 7 days after inoculation (Fig. 3B). Compared to the negative control group (176.1 U/ml), the free-ceramide group slightly increased the IgM antibody level to 528.3 U/ml, and the LNP-ceramide group increased rapidly to 2279.7 U/ml. The IgM antibody level was higher in the LNP-DMG group (1373.5 U/ml) than in the free-DMG group (578.8 U/ml). As a result, it was established that PEG lipid conjugated on the mRNA/LNP’s surface, rather than free-PEG lipid, was recognized by the spleen’s marginal B cells, causing them to generate and secrete anti-PEG IgM. Additionally, we hypothesized that PEG-attached LNPs induced the production of more anti-PEG antibodies than free-PEG lipids because they are recognized as large molecular weight PEGs. Therefore, large free PEG 35k was injected at the same molar amount (Fig. 3A). After 7 days, the free-PEG 35k group (1737.5 U/ml) had a higher antibody level than the free-DMG PEG and free-ceramide PEG groups and a similar level of antibody to the PEG-conjugated LNP group (Fig. 3B). To examine how the molecular weight of PEG affects the antibody formation and the protein expression, the 10k and 35k free PEGs were injected into mice (Fig. S3). The mole of free PEGs was the same as DMG 1.1% LNP. One week later, hEPO mRNA encapsulating DMG 1.1% LNP was injected to all groups. As a result, the antibody concentration of the LNP group has the same level as the free-PEG 35k group. The free-PEG 10k group was lower than the other group, which is consistent with our rationale. In contrast, the hEPO level of the free-PEG 10k group was higher than other groups.

### The initial immunogenicity of LNPs does not affect the ABC phenomenon

**Fig. 4.**
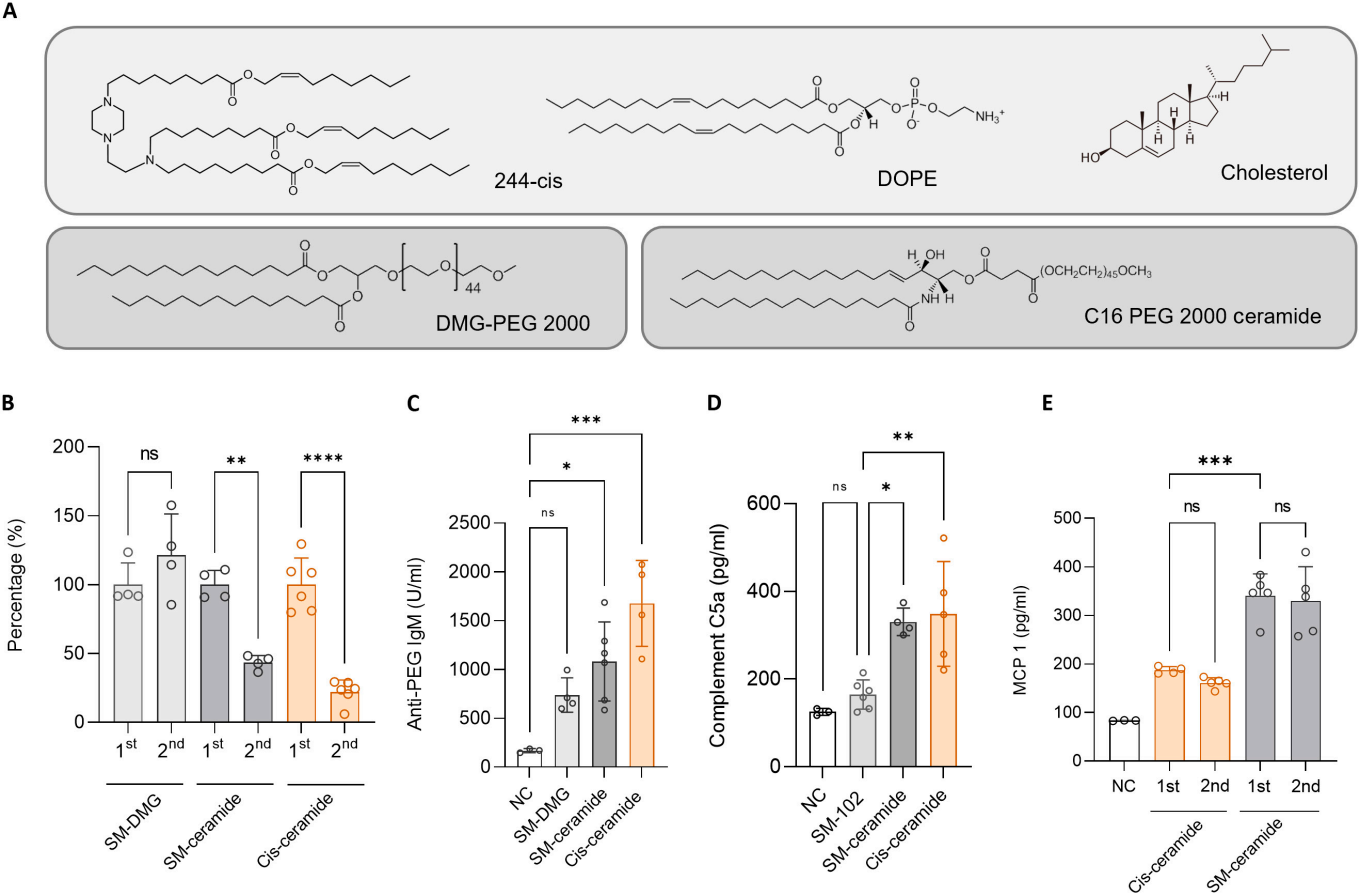
Evaluation of the repeated administration of 244-cis LNP and evaluation of the initial immunity to LNP. (A) Components of 244-cis LNP. 244-cis ionizable lipid is a piperazine-based biodegradable ionizable lipid that has a different structure from SM-102. In addition to ionizable lipids, 244-cis LNP contains DOPE (1,2-dioleoyl-sn-glycerol-3-phosphoethanolamine), cholesterol, and C16 PEG. (B) Evaluation of protein expression following the repeated administration of hEPO mRNA/LNP. Protein expression following two administrations of SM-102 LNP and 244-cis LNP encapsulated with hEPO mRNA at 7-day intervals was converted to a percentage based on the first administration. Protein expression decreased during the second administration of the LNP groups containing C16 PEG 2000 ceramide PEG lipid. (C) Concentration of anti-PEG IgM 1 week after LNP administration. It was confirmed that the antibody concentration of C16 PEG ceramide LNP was higher than that of DMG-PEG-LNP. (D) Complement C5a concentration after a second administration of LNP. Complement activity appears to be increasing as the complement C5a concentration in the ceramide LNP group slightly increases. (E) Initial immunogenicity when repeated doses of LNP were administered. After the first and second administrations, the level of monocyte chemoattractant protein (MCP-1) was evaluated after the initial administration of 244-cis LNP and SM-102 LNP. There was no difference in the amount of MCP-1 secretion between the first and second administrations of LNP, and the SM-ceramide LNP group showed higher levels than the cis-ceramide group.

A new ionizable lipid 244-cis was tested and compared to SM-102 LNP to validate the effects of repeated administration. The 244-cis ionizable lipid is a piperazine-based biodegradable ionizable lipid with a structure different from SM-102 (Fig. 4A) [34]. 244-cis LNP is composed of DOPE (1,2-dioleoyl-sn-glycero-3-phosphoethanolamine), cholesterol, and C16 PEG ceramide (Fig. 4A). The repeated administration schedules for these two LNPs were performed as shown in Fig. 2B. This time, we compared SM-102 LNP incorporated DMG-PEG and C16 PEG ceramide and 244-cis LNP with C16 PEG ceramide. The physical properties of LNPs are shown in Table S1. Protein expression after the second administration of mRNA/LNP was converted into a percentage based on the mRNA expression of the first administration (Fig. 4B). Figure S1 provides details of the hEPO concentration. The SM-102 LNP with DMG-PEG maintained the protein expression level of the first administration (121.3%), whereas the 244-cis LNP and SM-102 LNP with C16 PEG ceramide decreased rapidly to 22.2% and 43.4%, respectively. Anti-PEG IgM (Fig. 4C) and complement C5a (Fig. 4D) concentrations were also higher in C16 PEG ceramide-conjugated LNP. To confirm whether the initial immunogenicity of LNP affected repeated administrations, the levels of monocyte chemoattractant protein (MCP-1) were analyzed after the initial administration of 244-cis LNP and SM-102 LNP introduced C16 PEG ceramide (Fig. 4E). As a result of investigating the MCP-1 concentration by blood sampling 3 h after the intravenous injection of 0.1 mg/kg of hEPO mRNA, SM-102 C16 PEG ceramide LNP (340.8 and 329.8 pg/ml) had a higher level of MCP-1 secretion than 244-cis LNP (187.0 and 160.8 pg/ml). Furthermore, the initial immunogenicity did not change following the first and second administrations. Therefore, it was confirmed that the initial immunogenicity of LNPs does not affect repeated administrations.

### PEG lipid optimization for repeated doses of 244-cis LNP

**Fig. 5.**
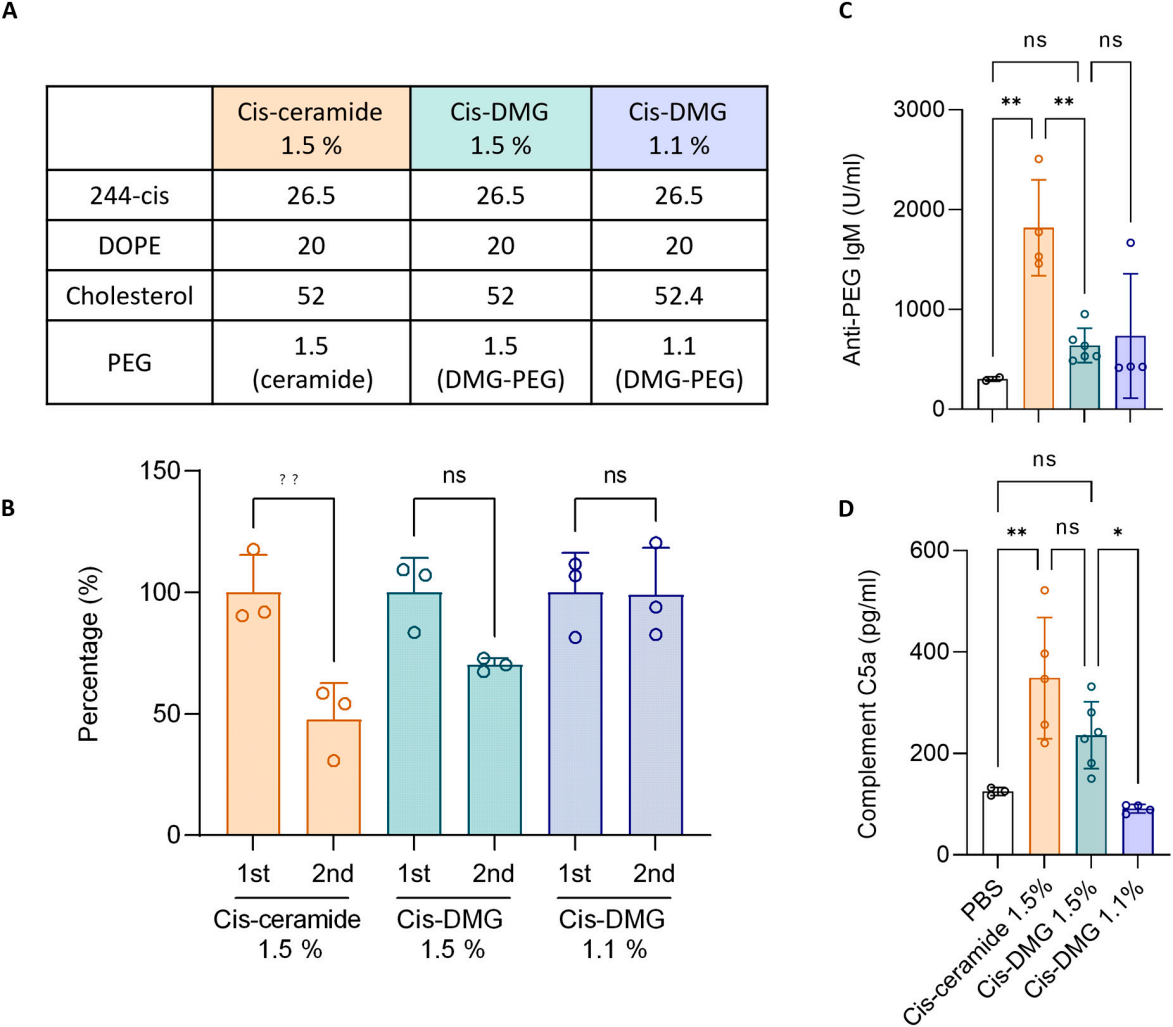
PEG lipid optimization of 244-cis LNP. (A) Molar ratio of three types of 244-cis LNP and PEG lipids. (B) Evaluation of protein expression following the repeated administration of hEPO mRNA/LNP. Protein expression following two administrations of 244-cis LNPs encapsulated with hEPO mRNA at 7-day intervals was converted to a percentage based on the first administration. Protein expression decreased during the second administration of the cis-ceramide 1.5% group and cis-DMG 1.5% group. (C) Concentration of anti-PEG IgM 1 week after LNP administration. It was confirmed that the antibody concentration of C16 PEG ceramide LNP was higher than that of DMG-PEG-LNP. (D) Complement C5a concentration after a second administration of LNP. Complement activity decreased in the following order: cis-ceramide 1.5%, cis-DMG 1.5%, and cis-DMG 1.1%.

Using the prior study as an indication, repeated doses of 244-cis LNP were administered by altering the PEG lipid. Protein expression was compared after the intravenous injection of hEPO mRNA encapsulated LNPs at weekly intervals (Fig. 2B). Figure S1 provides details of the hEPO concentration. When C16 PEG ceramide was used, the protein expression of the second administration decreased by 47.7% compared to the first administration, but it was confirmed that it was restored to 70.2% when changed to DMG-PEG (Fig. 5B). However, because it did not recover to the level of the first dose, the molar ratio of DMG-PEG, which was 1.5%, was lowered to 1.1%, and two doses were administered (Fig. 5A). A molar ratio of 1.1% is the minimum ratio for PEG. If it falls further than that (1.0%), the encapsulation efficiency will fall below 80% (Table S1). As a result, DMG-PEG 1.1% LNP recovered 99.0% of the first dose (Fig. 5B). Anti-PEG IgM and complement C5a levels were measured as in previous investigations (Fig. 2B). C16 PEG ceramide LNP (1817.7 U/ml) had considerably greater antibody levels than DMG-PEG 1.5% LNP (639.6 U/ml) and DMG-PEG 1.1% LNP (734.5 U/ml) (Fig. 5C). The complement C5a level was slightly higher for C16 PEG ceramide LNP (348.7 U/ml) than for DMG-PEG 1.5% LNP (236.1 U/ml), and DMG-PEG 1.5% LNP had a significantly higher concentration of complement C5a than DMG-PEG 1.1% LNP (91.2 U/ml) (Fig. 5D). As a result, the structure and molar ratio of PEG lipid were crucial for protein expression in successive administrations of LNPs. The results of hEPO 0.1 mg/kg and analysis by time point have been added to Fig. S2. Ceramide 1.5% failed to maintain the protein expression level at the second administration, whereas DMG 1.1% LNP maintained the protein expression level up to the first administration level. The area under the curve (AUC) value of ceramide 1.5% decreased significantly at the second administration, and the total value decreased by half compared to DMG 1.1%.

### Repeated dose evaluation of fLuc mRNA expression when PEG lipid is optimized

**Fig. 6.**
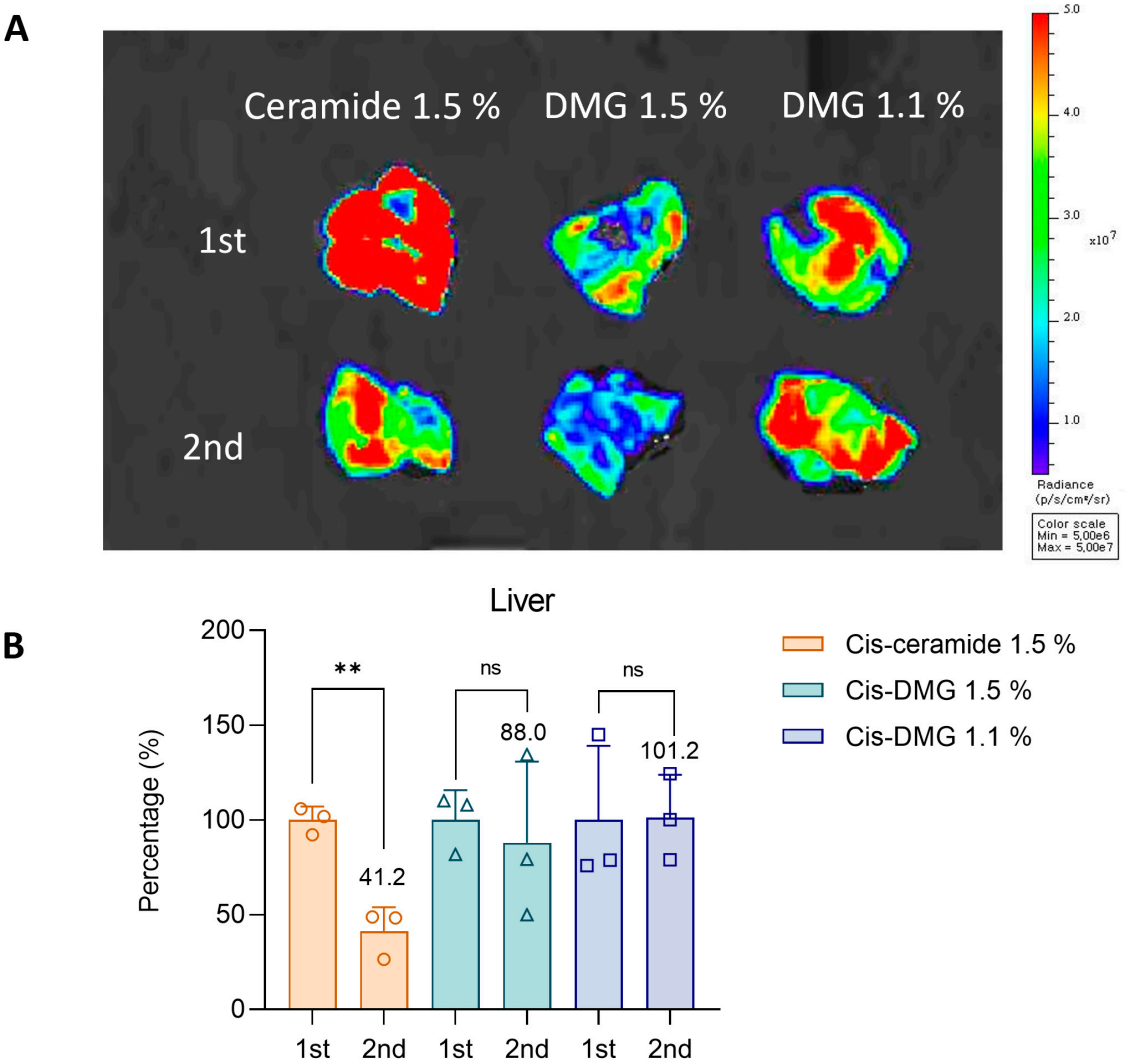
Repeated dose evaluation of fLuc mRNA expression (A) IVIS image of the liver following the repeated administration of fLuc mRNA/LNP. Protein expression following two administrations of 244-cis LNPs encapsulated with fLuc mRNA at 7-day intervals was visualized with an IVIS Lumina In vivo Imaging System. Protein expression decreased during the second administration of the cis-ceramide 1.5% group and cis-DMG 1.5% group. No decrease in bioluminescence values was observed in the cis-DMG 1.1% group. (B) The bioluminescence following the second administration of LNP was converted to a percentage based on the first administration. In the case of the cis-ceramide 1.5% group, the luminescence values in the liver were significantly lower compared to the first administration. In the case of cis-DMG 1.5% LNP, the decrease was relatively small. No decrease in bioluminescence values was observed in the cis-DMG 1.1% group.

When the PEG of the 244-cis LNP was optimized, the change in fLuc mRNA expression in the liver after repeated administrations was analyzed. Repeated administrations were evaluated at weekly intervals. One LNP group was divided into two groups, and half were injected 1 week earlier to generate anti-PEG IgM. One week later, both groups were injected and differences in repeated administrations were investigated. The bioluminescence of the first and second dose groups was observed 6 h after the intravenous administration at 0.1 mg/kg fLuc mRNA/LNPs. The liver was extracted and bioluminescence was visualized (Fig. 6A), and the first and second administrations were compared using quantified values of the IVIS Lumina In vivo Imaging System (Fig. 6B). The second dose was expressed as a relative percentage compared to the first dose. Quantitative bioluminescence values of the liver, spleen, and lung are presented in Fig. S4. By IVIS, it was discovered that the cis-ceramide LNP group had decreased protein expression compared with the cis-DMG LNP group after the second dosage. For the cis-ceramide 1.5% group, the luminescence values in the liver were significantly lower (41.2%) compared to the first administration. For cis-DMG 1.5% LNP, the decrease was relatively small (88.0%). No decrease in bioluminescence values was observed in the cis-DMG 1.1% group (101.2%). Therefore, when the PEG lipid and composition of LNPs were changed, there was a difference in organ biodistribution and protein expression after the first and second administrations.

### Repeated dose evaluation of Cre mRNA gene editing when PEG lipid is optimized

**Fig. 7.**
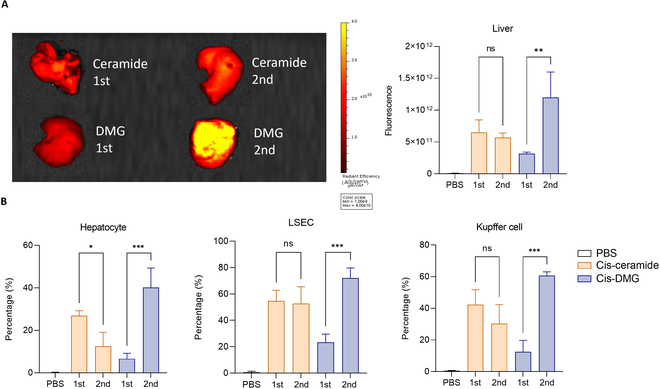
Repeated dose evaluation of Cre mRNA gene editing. (A) IVIS image and fluorescence values of the liver following the repeated administration of Cre mRNA/LNP. When checking tdTomato fluorescence imaging, there was no significant difference in fluorescence values between the first and second doses of C16 PEG ceramide 1.5% LNP. DMG-PEG 1.1% LNP showed an overlap between primary and secondary gene editing efficiencies. (B) Within the livers, cell type-specific Cre-mediated recombination was evaluated and single cells were extracted and examined by flow cytometry. All LNP groups showed transfection efficiency similar to (A) in hepatocytes, LSEC, and Kupffer cells.

We evaluated how the distribution of liver cells changed when the PEG of 244-cis LNP was optimized (DMG-PEG 1.1%) and how the effect of gene editing with repeated administrations changed. After the encapsulation of Cre recombinase mRNA (Cre mRNA) of 244-cis LNP, repeated administrations were evaluated at weekly intervals into tdTomato transgenic mice. One LNP group was divided into two groups, and half were injected 1 week earlier to generate anti-PEG IgM. One week later, both groups were injected and differences in repeated administrations were investigated. The first and second administration evaluations were analyzed by in vivo imaging (Fig. 7A) and flow cytometry (Fig. 7B) 48 h after intravenous injection at a concentration of 0.01 mg/kg. Fluorescence images and quantitative data of organs are shown in Fig. S5. There was no significant variation in fluorescence values between the first and second administrations of C16 PEG ceramide LNP, as validated by tdTomato fluorescence imaging. Optimized LNP showed overlapping efficiencies of primary and secondary gene editing (Fig. 7A). When the second dose of C16 PEG ceramide LNP was administrated, the expression of second Cre mRNA seemed to decrease rapidly due to the ABC phenomenon. However, in the case of DMG-PEG LNP, Cre recombinase of the first and second administrations was normally expressed, confirming that the gene editing effect had accumulated.

Then, within the livers, cell type-specific Cre-mediated recombination was evaluated, and single cells were extracted and examined by flow cytometry (Fig. 7B). Transfection efficiencies following repeated administrations were compared in hepatocytes, liver sinusoidal endothelial cells (LSECs), and Kupffer cells, respectively. Similar to tdTomato fluorescence, C16-PEG ceramide LNP showed similar efficiency to that of the first round without overlapping gene editing effects in all cells during the first and second administrations. However, DMG-PEG LNP showed a significant increase after the second administration rather than after the first administration in the three liver cell types, confirming that the gene editing effect appeared to have an additional effect with repeated administrations.

## Conclusion

In this study, we investigated the influence of LNP formulations with varying PEG lipids and ionizable lipids on the repeated administration of mRNA-loaded LNPs (mRNA/LNP). Our investigations demonstrated that ionizable lipids exhibited negligible effects on the outcomes of repeated dosages. In contrast, the lipid structure and molar ratio of PEG lipids exerted a substantial influence on the protein expression of mRNA/LNP. It became apparent that PEG molecules linked to LNPs, as opposed to free PEG, played a pivotal role in the generation of anti-PEG IgM antibodies. Within the biological milieu, PEG-incorporated LNPs assumed the character of large PEG entities, potentially provoking antibody production. Upon subsequent injections, this phenomenon culminated in a diminution of protein expression by mRNA/LNP. Specifically, successive administrations of firefly luciferase (fLuc) mRNA caused a large decline in luminescent expression within the liver. The assessment of gene editing effects following repeated administrations yielded variations contingent upon the specific PEG lipid used.

Notably, our investigation revealed that an increase in the lipid tail length of the PEG lipid corresponded to a reduction in PEG shedding rates and an escalation in anti-PEG IgM production, ultimately triggering the ABC phenomenon. This observation confirmed previous studies of PEG-NPs [33]. Upon exposure to blood, PEG lipid shedding commenced within the LNP, with the process culminating in the spleen. Within the splenic marginal zone, B cells are responsible for the synthesis of antibodies. Consequently, it is suggested that LNPs exhibiting diminished PEG shedding would augment the synthesis of anti-PEG IgM. From this perspective, C14 DMG-PEG 2000, characterized by a shorter lipid tail length, exhibited a higher likelihood of shedding within LNPs compared to C16 PEG 2000 ceramide.

This study also provided a comparative investigation of the protein expression efficiency exhibited by C16 PEG ceramide-incorporated LNPs as opposed to DMG-PEG LNPs during their initial administration. The results revealed that the first administration of C16 PEG ceramide-conjugated LNPs yielded relatively higher protein expression efficiency in comparison to DMG-PEG LNPs. However, a noteworthy decline in the efficacy of C16 PEG ceramide LNPs was observed upon their second administration compared to the first. This observation is significant when delineating the choice between single-dose or repeat-dose regimens for mRNA/LNP treatments. Moreover, the selection of the PEG lipid to be incorporated into LNPs may depend on the objective of achieving maximal therapeutic efficacy by a single dose or sustaining therapeutic effects through repeated administrations.

The two examined lipid constituents, 244-cis and SM-102, exhibit structural disparities and distinct formulation ratios. The SM-102 lipid features a single tertiary amine structure, whereas the 244-cis lipid has a piperazine structure with three tertiary amines, imparting it with a bulkier conformation. This structural dissimilarity implies potential variations in the lipid content and ratio within the LNPs. The optimized molar ratio for 244-cis LNPs was determined as 244-cis:DOPE:cholesterol:C16-PEG ceramide = 26.5:20:52:1.5, whereas the ratio for SM-102 LNPs was SM-102:DSPC:cholesterol:DMG-PEG 2000 = 50:10:38.5:1.5. Notably, the ionizable lipid ratio of 244-cis LNPs was lower than that of SM-102 LNPs. This resulted in the greater use of other lipid components in LNP formulations such as helper lipids, cholesterol, and PEG lipids. For the helper lipids, SM-102 LNPs used DSPC, whereas DOPE was the chosen lipid for 244-cis LNPs. Our findings suggest that the phospholipid head group exhibits a stronger binding affinity with ionizable lipids when characterized by a phosphoethanolamine (PE) structure as opposed to a phosphocholine (PC) structure [39]. It is likely that the bulky nature of DSPC may introduce steric hindrance when interacting with 244-cis. Indeed, our prior investigations demonstrated a significant reduction in RNA encapsulation efficiency when the compositional components and molar ratios of SM-102 LNPs were adapted to 244-cis.

In conclusion, this study systematically examined the efficacy of mRNA/LNP in the liver during repeated administrations. As ongoing LNP research extends its focus to include extrahepatic organs [40,41], it is necessary to emphasize the need for continued investigations. This highlights the importance of follow-up research to assess the feasibility and optimization of repeated administration strategies for LNPs designed to target extrahepatic tissues and to accommodate various clinical applications. Furthermore, it is essential to extend the evaluation and formulation optimization processes beyond murine models to nonhuman primates possessing more intricate immune systems. In light of these considerations, the findings of this study serve to emphasize the critical nature of formulation optimization with regard to ionizable lipids and the consequential significance of carefully determining the PEG lipid and its ratio for achieving successful repeated administration of mRNA therapeutics.

## Data Availability

The data that support the findings of this study are available from the corresponding authors upon reasonable request.

## References

[1] Swetha K, Kotla NG, Tunki L, Jayaraj A, Bhargava SK, Hu H, Bonam SR, Kurapati R. Recent advances in the lipid nanoparticle-mediated delivery of mRNA vaccines. Vaccines. 2023;11(3): Article 658. 36992242 10.3390/vaccines11030658 PMC10059764

[2] Gote V, Bolla PK, Kommineni N, Butreddy A, Nukala PK, Palakurthi SS, Khan W. A comprehensive review of mRNA vaccines. Int J Mol Sci. 2023;24(3): Article 2700. 36769023 10.3390/ijms24032700 PMC9917162

[3] Kim M, Jeong M, Hur S, Cho Y, Park J, Jung H, Seo Y, Woo HA, Nam KT, Lee K, et al. Engineered ionizable lipid nanoparticles for targeted delivery of RNA therapeutics into different types of cells in the liver. Sci Adv. 2021;7(9):eabf4398. 33637537 10.1126/sciadv.abf4398 PMC7909888

[4] Han X, Zhang H, Butowska K, Swingle KL, Alameh MG, Weissman D, Mitchell MJ. An ionizable lipid toolbox for RNA delivery. Nat Commun. 2021;12(1):7233. 34903741 10.1038/s41467-021-27493-0 PMC8668901

[5] Kumar V, Qin J, Jiang Y, Duncan RG, Brigham B, Fishman S, Nair JK, Akinc A, Barros SA, Kasperkovitz PV. Shielding of lipid nanoparticles for siRNA delivery: Impact on physicochemical properties, cytokine induction, and efficacy. Mol Ther Nucleic Acids. 2014;3(11): Article e210. 25405467 10.1038/mtna.2014.61 PMC4459547

[6] Akinc A, Goldberg M, Qin J, Dorkin JR, Gamba-Vitalo C, Maier M, Jayaprakash KN, Jayaraman M, Rajeev KG, Manoharan M, et al. Development of lipidoid–siRNA formulations for systemic delivery to the liver. Mol Ther. 2009;17(5):872–879. 19259063 10.1038/mt.2009.36 PMC2835134

[7] Houseley J, Tollervey D. The many pathways of RNA degradation. Cell. 2009;136(4):763–776. 19239894 10.1016/j.cell.2009.01.019

[8] Kristen AV, Ajroud-Driss S, Conceição I, Gorevic P, Kyriakides T, Obici L. Patisiran, an RNAi therapeutic for the treatment of hereditary transthyretin-mediated amyloidosis. Neurodegener Dis Manag. 2019;9(1):5–23. 30480471 10.2217/nmt-2018-0033

[9] Falsey AR, Frenck RW Jr, Walsh EE, Kitchin N, Absalon J, Gurtman A, Lockhart S, Bailey R, Swanson KA, Xu X, et al. SARS-CoV-2 neutralization with BNT162b2 vaccine dose 3. N Engl J Med. 2021;385(17):1627–1629. 34525276 10.1056/NEJMc2113468 PMC8461567

[10] Baden LR, el Sahly HM, Essink B, Kotloff K, Frey S, Novak R, Diemert D, Spector SA, Rouphael N, Creech CB, et al. Efficacy and safety of the mRNA-1273 SARS-CoV-2 vaccine. N Engl J Med. 2021;384(5):403–416. 33378609 10.1056/NEJMoa2035389 PMC7787219

[11] Chen C-Y, Tran DM, Cavedon A, Cai X, Rajendran R, Lyle MJ, Martini PGV, Miao CH. Treatment of hemophilia A using factor VIII messenger RNA lipid nanoparticles. Mol Ther Nucleic Acids. 2020;20:534–544. 32330871 10.1016/j.omtn.2020.03.015 PMC7178004

[12] DeRosa F, Guild B, Karve S, Smith L, Love K, Dorkin JR, Kauffman KJ, Zhang J, Yahalom B, Anderson DG, et al. Therapeutic efficacy in a hemophilia B model using a biosynthetic mRNA liver depot system. Gene Ther. 2016;23(10):699–707. 27356951 10.1038/gt.2016.46 PMC5059749

[13] Cheng Q, Wei T, Jia Y, Farbiak L, Zhou K, Zhang S, Wei Y, Zhu H, Siegwart DJ. Dendrimer-based lipid nanoparticles deliver therapeutic FAH mRNA to normalize liver function and extend survival in a mouse model of hepatorenal tyrosinemia type I. Adv Mater. 2018;30(52): Article e1805308. 30368954 10.1002/adma.201805308

[14] Jiang L, Berraondo P, Jericó D, Guey LT, Sampedro A, Frassetto A, Benenato KE, Burke K, Santamaría E, Alegre M, et al. Systemic messenger RNA as an etiological treatment for acute intermittent porphyria. Nat Med. 2018;24(12):1899–1909. 30297912 10.1038/s41591-018-0199-z

[15] Roseman DS, Khan T, Rajas F, Jun LS, Asrani KH, Isaacs C, Farelli JD, Subramanian RR. G6PC mRNA therapy positively regulates fasting blood glucose and decreases liver abnormalities in a mouse model of glycogen storage disease 1a. Mol Ther. 2018;26(3):814–821. 29428299 10.1016/j.ymthe.2018.01.006 PMC5910675

[16] Finn JD, Smith AR, Patel MC, Shaw L, Youniss MR, van Heteren J, Dirstine T, Ciullo C, Lescarbeau R, Seitzer J, et al. A single administration of CRISPR/Cas9 lipid nanoparticles achieves robust and persistent in vivo genome editing. Cell Rep. 2018;22(9):2227–2235. 29490262 10.1016/j.celrep.2018.02.014

[17] Villiger L, Rothgangl T, Witzigmann D, Oka R, Lin PJC, Qi W, Janjuha S, Berk C, Ringnalda F, Beattie MB, et al. In vivo cytidine base editing of hepatocytes without detectable off-target mutations in RNA and DNA. Nat Biomed Eng. 2021;5(2):179–189. 33495639 10.1038/s41551-020-00671-z PMC7610981

[18] Jiang T, Henderson JM, Coote K, Cheng Y, Valley HC, Zhang XO, Wang Q, Rhym LH, Cao Y, Newby GA, et al. Chemical modifications of adenine base editor mRNA and guide RNA expand its application scope. Nat Commun. 2020;11(1):1979. 32332735 10.1038/s41467-020-15892-8 PMC7181807

[19] Shimizu T, Mima Y, Hashimoto Y, Ukawa M, Ando H, Kiwada H, Ishida T. Anti-PEG IgM and complement system are required for the association of second doses of PEGylated liposomes with splenic marginal zone B cells. Immunobiology. 2015;220(10):1151–1160. 26095176 10.1016/j.imbio.2015.06.005

[20] Kozma GT, Mészáros T, Vashegyi I, Fülöp T, Örfi E, Dézsi L, Rosivall L, Bavli Y, Urbanics R, Mollnes TE, et al. Pseudo-anaphylaxis to polyethylene glycol (PEG)-coated liposomes: Roles of anti-PEG IgM and complement activation in a porcine model of human infusion reactions. ACS Nano. 2019;13(8):9315–9324. 31348638 10.1021/acsnano.9b03942

[21] Zhao Y, Wang L, Yan M, Ma Y, Zang G, She Z, Deng Y. Repeated injection of PEGylated solid lipid nanoparticles induces accelerated blood clearance in mice and beagles. Int J Nanomedicine. 2012;7:2891–2900. 22745552 10.2147/IJN.S30943 PMC3383289

[22] Grenier P, Viana IMO, Lima EM, Bertrand N. Anti-polyethylene glycol antibodies alter the protein corona deposited on nanoparticles and the physiological pathways regulating their fate in vivo. J Control Release. 2018;287:121–131. 30138715 10.1016/j.jconrel.2018.08.022

[23] Wan C, Allen TM, Cullis PR. Lipid nanoparticle delivery systems for siRNA-based therapeutics. Drug Deliv Transl Res. 2014;4(1):74–83. 25786618 10.1007/s13346-013-0161-z

[24] Besin G, Milton J, Sabnis S, Howell R, Mihai C, Burke K, Benenato KE, Stanton M, Smith P, Senn J, et al. Accelerated blood clearance of lipid nanoparticles entails a biphasic humoral response of B-1 followed by B-2 lymphocytes to distinct antigenic moieties. ImmunoHorizons. 2019;3(7):282–293. 31356158 10.4049/immunohorizons.1900029

[25] Zhang P, Sun F, Liu S, Jiang S. Anti-PEG antibodies in the clinic: Current issues and beyond PEGylation. J Control Release. 2016;244(Pt B):184–193. 27369864 10.1016/j.jconrel.2016.06.040 PMC5747248

[26] Mohamed M, Abu Lila AS, Shimizu T, Alaaeldin E, Hussein A, Sarhan HA, Szebeni J, Ishida T. PEGylated liposomes: Immunological responses. Sci Technol Adv Mater. 2019;20(1):710–724. 31275462 10.1080/14686996.2019.1627174 PMC6598536

[27] Kozma GT, Shimizu T, Ishida T, Szebeni J. Anti-PEG antibodies: Properties, formation, testing and role in adverse immune reactions to PEGylated nano-biopharmaceuticals. Adv Drug Deliv Rev. 2020;154–155:163–175. 10.1016/j.addr.2020.07.024 32745496

[28] Wang F, Wu Y, Zhang J, Wang H, Xie X, Ye X, Peng D, Chen W. Induction of cytochrome P450 involved in the accelerated blood clearance phenomenon induced by PEGylated liposomes in vivo. Drug Metab Dispos. 2019;47(4):364–376. 30674617 10.1124/dmd.118.085340

[29] Liang K, Wang L, Su Y, Liu M, Feng R, Song Y, Deng Y. Comparison among different “revealers” in the study of accelerated blood clearance phenomenon. Eur J Pharm Sci. 2018;114:210–216. 29247685 10.1016/j.ejps.2017.12.010

[30] Su Y, Liu M, Xiong Y, Ding J, Liu X, Song Y, Deng Y. Effects of stability of PEGylated micelles on the accelerated blood clearance phenomenon. Drug Deliv Transl Res. 2019;9(1):66–75. 30378014 10.1007/s13346-018-0588-3

[31] Xu H, Ye F, Hu M, Yin P, Zhang W, Li Y, Yu X, Deng Y. Influence of phospholipid types and animal models on the accelerated blood clearance phenomenon of PEGylated liposomes upon repeated injection. Drug Deliv. 2015;22(5):598–607. 24524364 10.3109/10717544.2014.885998

[32] Jiao J, Jiao X, Wang C, Wei L, Wang G, Deng Y, Song Y. The contribution of PEG molecular weights in PEGylated emulsions to the various phases in the accelerated blood clearance (ABC) phenomenon in rats. AAPS PharmSciTech. 2020;21(8):300. 33140142 10.1208/s12249-020-01838-2

[33] Suzuki T, Suzuki Y, Hihara T, Kubara K, Kondo K, Hyodo K, Yamazaki K, Ishida T, Ishihara H. PEG shedding-rate-dependent blood clearance of PEGylated lipid nanoparticles in mice: Faster PEG shedding attenuates anti-PEG IgM production. Int J Pharm. 2020;588: Article 119792. 32827675 10.1016/j.ijpharm.2020.119792

[34] Kim M, Jeong M, Lee G, Lee Y, Park J, Jung H, Im S, Yang JS, Kim K, Lee H. Novel piperazine-based ionizable lipid nanoparticles allow the repeated dose of mRNA to fibrotic lungs with improved potency and safety. Bioeng Transl Med. 2023;8(6): Article e10556. 38023699 10.1002/btm2.10556 PMC10658549

[35] Estapé Senti M, de Jongh CA, Dijkxhoorn K, Verhoef JJF, Szebeni J, Storm G, Hack CE, Schiffelers RM, Fens MH, Boross P. Anti-PEG antibodies compromise the integrity of PEGylated lipid-based nanoparticles via complement. J Control Release. 2022;341:475–486. 34890719 10.1016/j.jconrel.2021.11.042

[36] Chen G, Yang Y, Gao X, Dou Y, Wang H, Han G, Wang R, Wang J, Wang L, Li X, et al. Blockade of complement activation product C5a activity using specific antibody attenuates intestinal damage in trinitrobenzene sulfonic acid induced model of colitis. Lab Investig. 2011;91(3):472–483. 21102504 10.1038/labinvest.2010.183

[37] Yang Q, Lai SK. Anti-PEG immunity: Emergence, characteristics, and unaddressed questions. Wiley Interdiscip Rev Nanomed Nanobiotechnol. 2015;7(5):655–677. 25707913 10.1002/wnan.1339 PMC4515207

[38] Cunnion KM, Krishna NK, Pallera HK, Pineros-Fernandez A, Rivera MG, Hair PS, Lassiter BP, Huyck R, Clements MA, Hood AF, et al. Complement activation and STAT4 expression are associated with early inflammation in diabetic wounds. PLOS ONE. 2017;12(1): Article e0170500. 28107529 10.1371/journal.pone.0170500 PMC5249255

[39] Ermilova I, Swenson J. DOPC versus DOPE as a helper lipid for gene-therapies: Molecular dynamics simulations with DLin-MC3-DMA. Phys Chem Chem Phys. 2020;22(48):28256–28268. 33295352 10.1039/d0cp05111j

[40] Cheng Q, Wei T, Farbiak L, Johnson LT, Dilliard SA, Siegwart DJ. Selective organ targeting (SORT) nanoparticles for tissue-specific mRNA delivery and CRISPR–Cas gene editing. Nat Nanotechnol. 2020;15(4):313–320. 32251383 10.1038/s41565-020-0669-6 PMC7735425

[41] LoPresti ST, Arral ML, Chaudhary N, Whitehead KA. The replacement of helper lipids with charged alternatives in lipid nanoparticles facilitates targeted mRNA delivery to the spleen and lungs. J Control Release. 2022;345:819–831. 35346768 10.1016/j.jconrel.2022.03.046 PMC9447088

