# Supplementary material for: Development of Lipid Nanoparticle Formulation for the Repeated Administration of mRNA Therapeutics
Source: Biomater Res. 2024 May 22;28:0017. doi: 10.34133/bmr.0017 (PMC11109479; doi:10.34133/bmr.0017)
Supplement: Supplementary 1 — Figs. S1 to S6 Table S1 [file bmr.0017.f1.docx]

**Supporting information**

**Yeji Lee^+^, Michaela Jeong, Gyeongseok Lee, Jeongeun Park, Hyein Jung, Seongeun Im and Hyukjin Lee***

*College of Pharmacy, Graduate School of Pharmaceutical Sciences, Ewha Womans University, Seoul 03760, Republic of Korea*

^*^Corresponding author.

Tel: +82-2-3277-3026

Fax: +82-2-3277-2851

E-mail address: hyukjin@ewha.ac.kr (Hyukjin Lee)

|  | Encapsulation efficiency (%) | Size (nm) | PDI |
| --- | --- | --- | --- |
| SM-102 DMG 1.5% | 92.3 ± 0.71 | 71.48 ± 4.33 | 0.106 ± 0.016 |
| SM-102 ceramide 1.5% | 92.9 ± 0.42 | 82.84 ± 4.86 | 0.079 ± 0.052 |
| 244-cis ceramide 1.5% | 91.7 ± 2.12 | 69.41 ± 2.71 | 0.077 ± 0.008 |
| 244-cis DMG 1.5% | 91.1 ± 1.70 | 68.29 ± 7.33 | 0.110 ± 0.018 |
| 244-cis DMG 1.1% | 89.5 ± 1.70 | 78.37 ± 6.19 | 0.074 ± 0.006 |
| 244-cis DMG 1.0% | 72.5 ± 3.54 | - | - |

**Table S1. Physical properties of LNPs** Encapsulation efficiency, size, and PDI index of LNPs used in this paper.

**
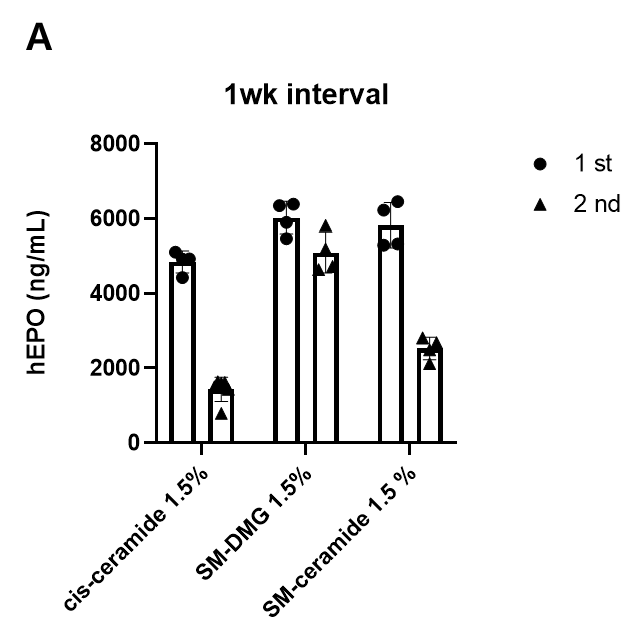
**

**
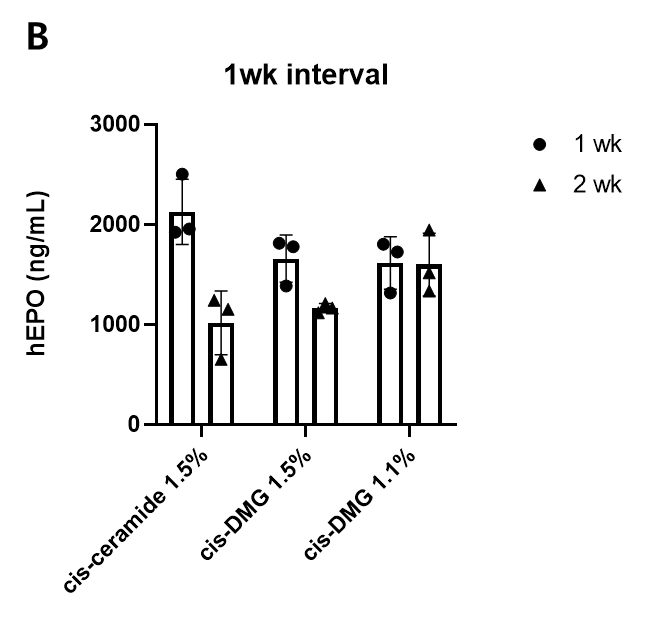
**

**Figure S1. hEPO concentration after the repeated administration of mRNA/LNP (A)** hEPO concentration of SM-DMG 1.5%, SM-ceramide 1.5%, and cis-ceramide 1.5% after the repeated administration of hEPO mRNA/LNP. **(B)** hEPO concentration of cis-ceramide, cis-DMG 1.5%, and cis-DMG 1.1% when hEPO mRNA/LNP was injected repeatedly.


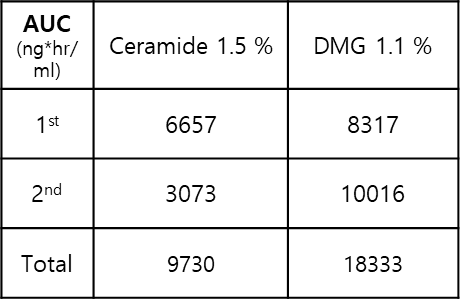


**Figure S2.** Protein expression and Area Under the Curve (AUC) after repeated administration of 0.1 mg/kg mRNA/LNP.


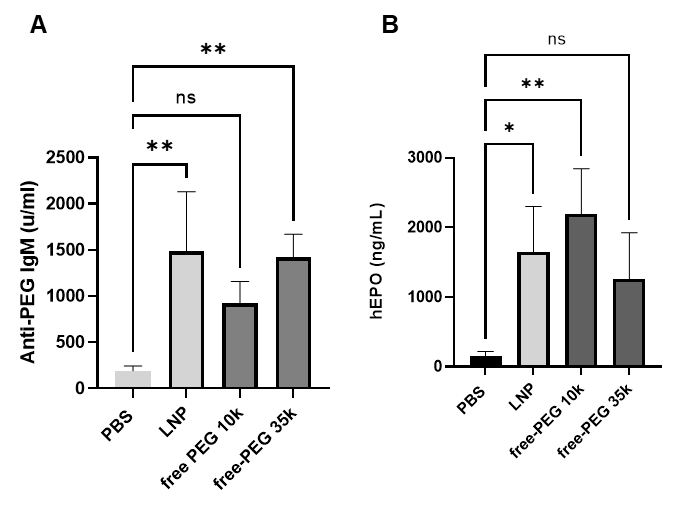


**Figure S3. Comparison of anti-PEG antibody formation between free PEG lipid, PEG incorporated LNP, and high molecular weight PEG (10k, 35k)** **(A)** Anti-PEG IgM antibody production of LNP and free PEG 10k, 35k and **(B)** resulting protein expression efficiency.

**
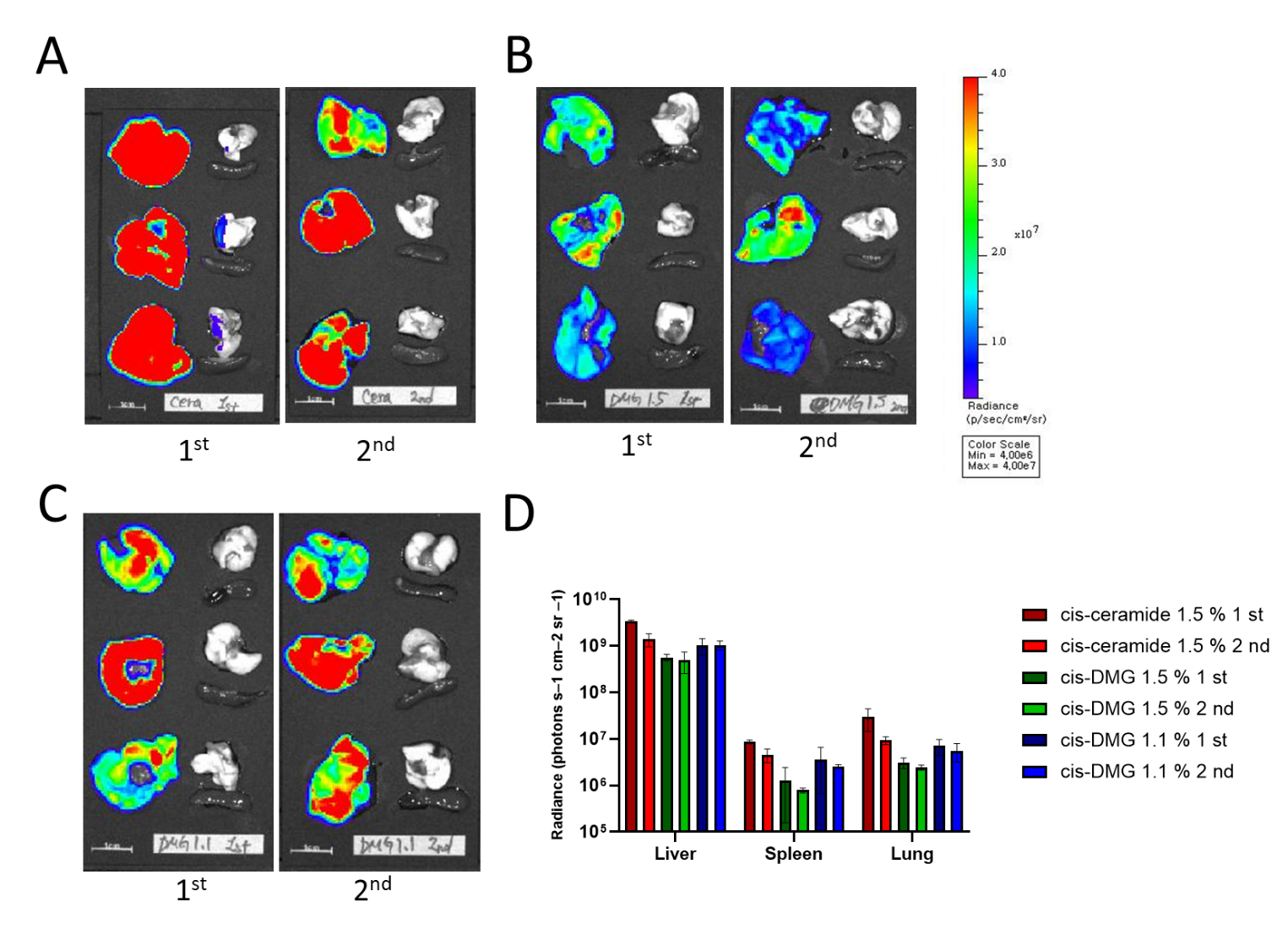
**

**Figure S4. Organ distribution of mRNA/LNP after repeated administration** IVIS image of organs following the repeated administration of **(A)** cis-ceramide 1.5% LNP, **(B)** cis-DMG 1.5 % LNP, and **(C)** cis-DMG 1.1 % LNP. **(D)** Quantitative bioluminescence values of the liver, spleen, and lung.

**
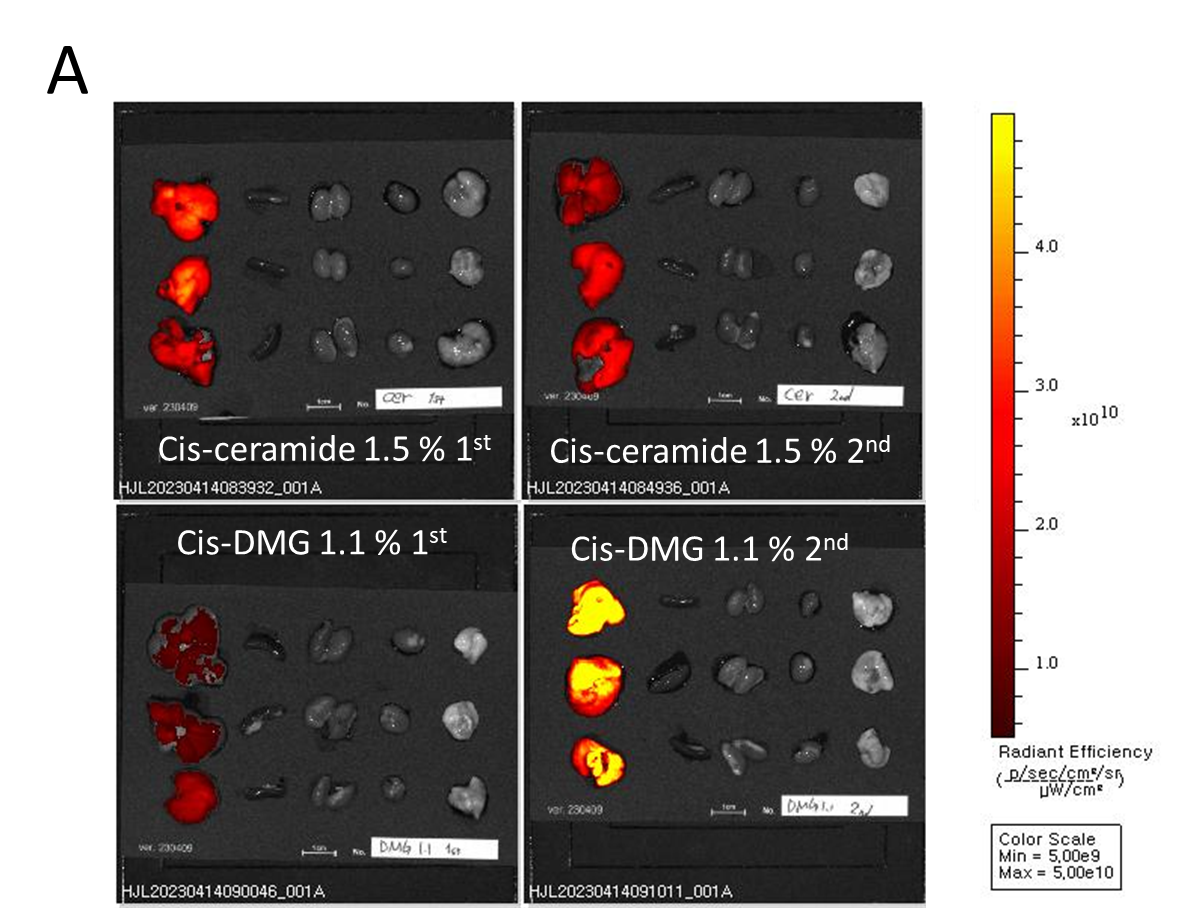
**

**
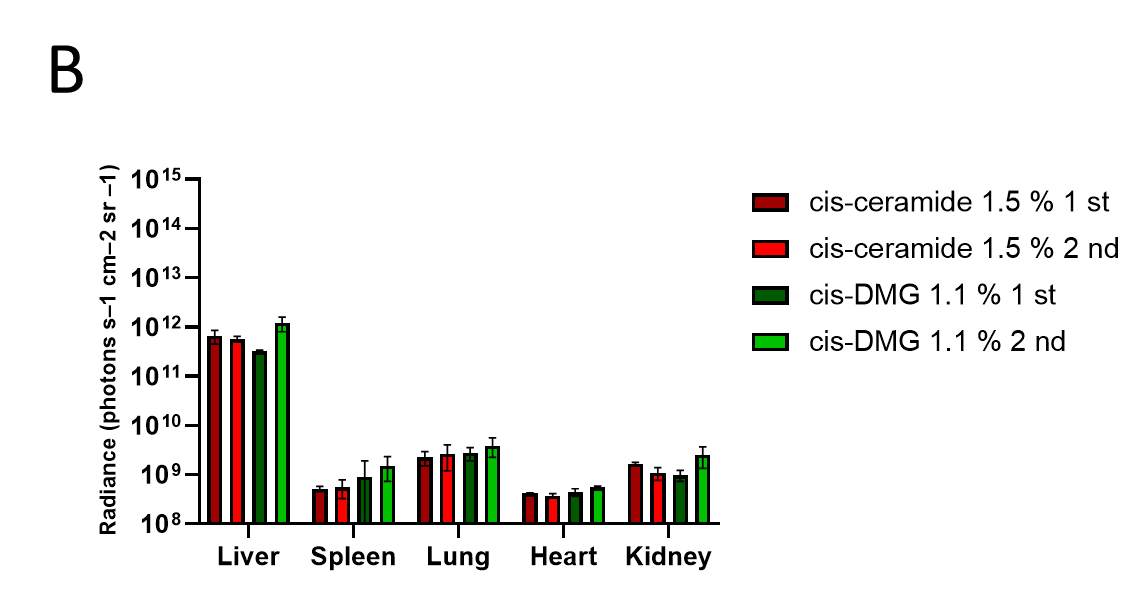
**

**Figure S5. Multiple dose evaluation of Cre mRNA gene editing (A)** IVIS images of organs following the repeated administration of cis-ceramide 1.5% LNP and cis-DMG 1.1% LNP. **(B)** Quantitative fluorescence values of all organs.

**
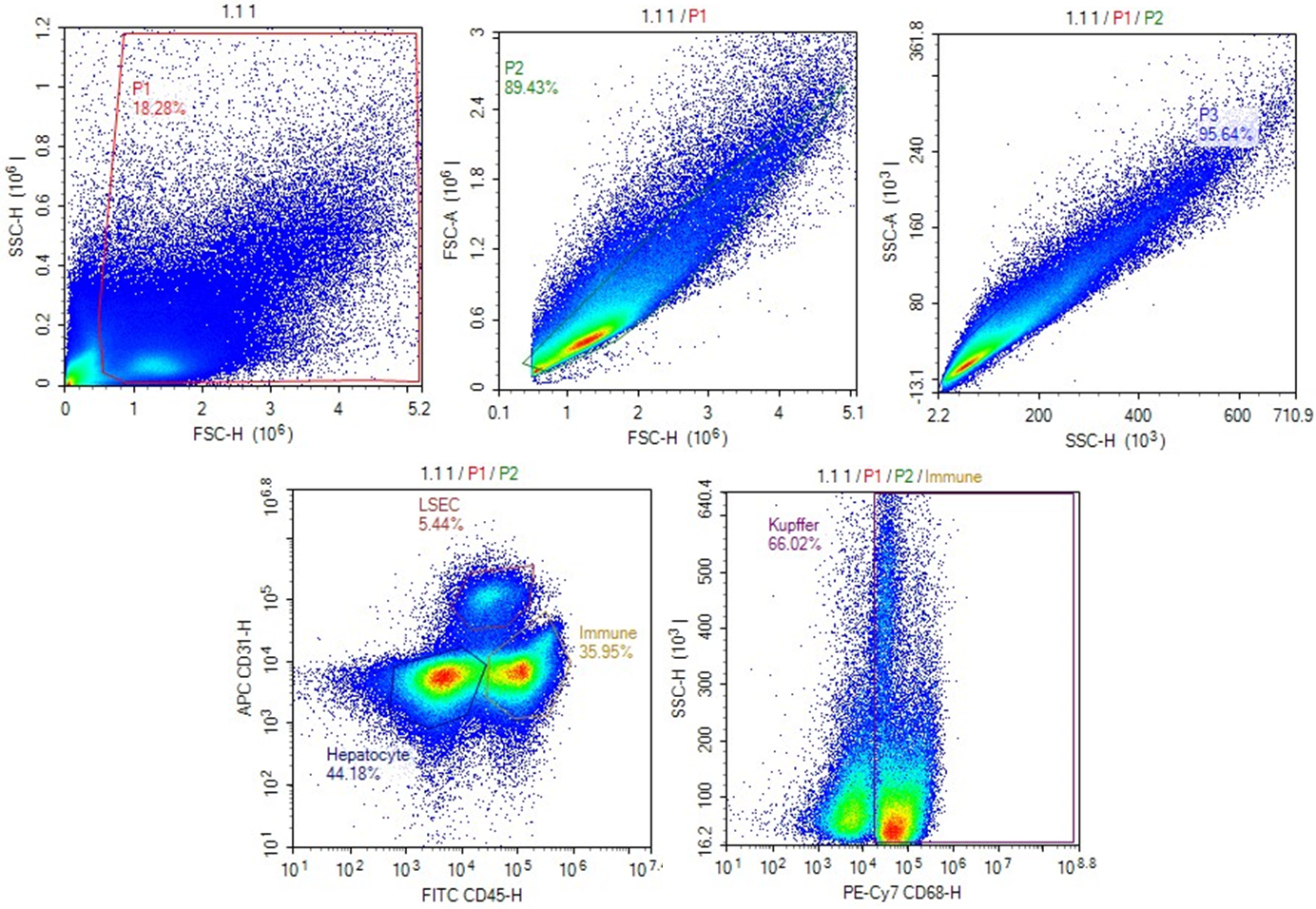
**

**Figure S6. Representative flow cytometry gating strategy of liver cells**
